# Food desert residence has limited impact on veteran fecal microbiome composition: a U.S. Veteran Microbiome Project study

**DOI:** 10.1128/msystems.00717-23

**Published:** 2023-10-24

**Authors:** Diana P. Brostow, Meghan Donovan, Molly Penzenik, Christopher E. Stamper, Talia Spark, Christopher A. Lowry, Suzanne L. Ishaq, Andrew J. Hoisington, Lisa A. Brenner

**Affiliations:** 1 VA Rocky Mountain Mental Illness Research Education and Clinical Center (MIRECC), Rocky Mountain Regional VA Medical Center (RMRVAMC), Aurora, Colorado, USA; 2 Department of Physical Medicine & Rehabilitation, University of Colorado Anschutz Medical Campus, Aurora, Colorado, USA; 3 Department of Psychiatry, University of Colorado Anschutz Medical Campus, Aurora, Colorado, USA; 4 Military and Veteran Microbiome Consortium for Research and Education, Aurora, Colorado, USA; 5 Department of Integrative Physiology, University of Colorado Boulder, Boulder, Colorado, USA; 6 Center for Neuroscience and Center for Microbial Exploration, University of Colorado Boulder, Boulder, Colorado, USA; 7 Center for Neuroscience, University of Colorado Anschutz Medical Campus, Aurora, Colorado, USA; 8 School of Food and Agriculture, University of Maine, Orono, Maine, USA; 9 Department of Systems Engineering & Management, Air Force Institute of Technology, Wright-Patterson AFB, Dayton, Ohio, USA; 10 Department of Neurology, University of Colorado Anschutz Medical Campus, Aurora, Colorado, USA; Oregon State University, Corvallis, Oregon, USA

**Keywords:** gut microbiome, food desert, diet, Veterans, mental health, PTSD, military, microbial composition, food access

## Abstract

Social and economic inequities can have a profound impact on human health, particularly on the development and progression of chronic disease. For military veterans, exposure to unique environments and circumstances may further impact their health. There continues to be limited work regarding the influence of mental health within the context of socioeconomic inequities. In this cross-sectional study, we hypothesized that veterans residing in food deserts (e.g., places in which there is a lack of access to sufficient and/or nutritious food) would have decreased gut microbial species (α-diversity), different microbiome community compositions, and poorer quality of diet and mental health compared to non-food desert residents. The fecal microbiome of 342 military veterans was sequenced, and microbiome diversity and community composition were evaluated. Although dietary quality and α-diversity did not significantly differ by food desert status, resident status (food desert versus non-food desert) accounted for a moderate influence on β-diversity (2.4%). Factors such as race and psychiatric diagnoses accounted for greater proportions of β-diversity influence (7% and 10%, respectively). Moreover, more participants with current post-traumatic stress disorder lived in food deserts (*P* < 0.04), and there were significantly more participants in the non-food desert group diagnosed with substance use disorders (*P* = 0.002) and current alcohol use disorder (*P* = 0.04). These findings suggest that living in a food desert, in combination with additional associated risk factors, may influence gut microbial diversity and composition. To increase ecological validity, researchers investigating the influence of inter-related biopsychosocial factors over time may benefit from adopting a life-course perspective.

**IMPORTANCE:**

Social and economic inequities can have a profound impact on human health. The inequities could result in alterations to the gut microbiome, an important factor that may have profound abilities to alter health outcomes. Moreover, the strong correlations between social and economic inequities have been long understood. However, to date, limited research regarding the microbiome and mental health within the context of socioeconomic inequities exists. One particular inequity that may influence both mental health and the gut microbiome is living in a food desert. Persons living in food deserts may lack access to sufficient and/or nutritious food and often experience other inequities, such as increased exposure to air pollution and poor access to healthcare. Together, these factors may confer a unique risk for microbial perturbation. Indeed, external factors beyond a food desert might compound over time to have a lasting effect on an individual’s gut microbiome. Therefore, adoption of a life-course approach is expected to increase the ecological validity of research related to social inequities, the gut microbiome, and physical and mental health.

## INTRODUCTION

Associations between social inequities and mental health are well established (1). In addition to frequently discussed factors such as poverty and inadequate access to healthcare, hygienic living spaces, safe neighborhood environments, and clean air (1) can promote and exacerbate psychiatric conditions such as depression, anxiety, and psychotic disorders (2). Nevertheless, even as the body of literature on mental health-gut microbiome interactions continues to expand (3, 4), there is a relative dearth of research examining these interactions within the context of social inequity.

One type of social inequity that has received increased attention is a lack of access to nutritious foods. “Food deserts” are areas in which residents have poor access to affordable or nutritious food (5). Food desert status is correlated with other adverse socioeconomic conditions such as poverty, unsafe living environments, and air pollution (6, 7). In terms of health behaviors and outcomes, residing in a food desert has been associated with a substantially increased risk of hospitalization, cardiovascular disease, and obesity (8, 9).

To date, food desert research has understandably focused on associations between factors of interest (e.g., cardiovascular disease) (10) and dietary quality, the latter of which is strongly correlated with socioeconomic inequities, health outcomes, and the gut microbiome (11–14). While diet-mental health associations (11, 13, 15) and diet-microbiome interactions are both reasonably well-researched, the body of literature on mental health and food deserts is sparse (16–18) and essentially nonexistent in relation to the gut microbiome. As of 2015, the U.S. Department of Agriculture (19) estimated that 12.8% of the population (approximately 39.4 million Americans) lived in low-income and low-food-access areas. At the same time, the national prevalence of psychiatric illness was significant, with nearly 20% (approximately 52 million Americans) struggling with mental disorders (20). Given these two substantial burdens and the lack of associated microbiome data, an examination of all three covariates is timely.

Military veterans may be a particularly unique cohort in regard to associations between food deserts, mental health, and the gut microbiome. Veterans experience a disproportionate burden of mental health conditions compared to their civilian counterparts (21). It is estimated that nearly a quarter of veterans who served in Iraq or Afghanistan have clinically significant post-traumatic stress disorder (PTSD) (22). While the socioeconomic and environmental factors that shape veterans’ health outcomes can vary by generation and combat era (e.g., veterans of the Vietnam era (23) compared to those who served after 9/11) (24), there is a large body of evidence establishing the complex financial, interpersonal, and employment-related challenges faced by veterans when attempting to reintegrate into civilian life (25). Veterans also experience unique exposures (e.g., military deployment, combat exposure, barrack-style living spaces), which may influence gut microbial composition even after completion of service. As a result, this population offers a unique lens through which to examine mental health and the gut microbiome in relation to food desert status.

An existing initiative in health equity and microbiome research is the Microbes and Social Equity Working Group, established in 2019 (26). The group’s goal is to promote incorporating microbiome and social equity factors into research studies to better understand the intersections between social determinates of health, microbes, and health outcomes. To achieve these objectives, health disparity research in host-microbiome interactions must move from peripheral discussion items to targeted areas of study (27). Specifically, there is increasing awareness of how social and economic inequities can shape the gut microbiome and subsequently impact the development of chronic health conditions (27, 28). Toward this end, we examined associations between food desert status, diet, mental health, demographic characteristics, military service history, and the gut microbiome. Our primary hypothesis was that veterans residing in food deserts would have lower α-diversity in the gut microbiome and poorer mental health than their non-food desert counterparts. We additionally hypothesized that veterans residing in food deserts would have an overall poorer quality of diet than their non-food desert counterparts.

## MATERIALS AND METHODS

### Participants

The U.S. Veteran Microbiome Project (US-VMP) is an ongoing longitudinal study. Veteran participants in the greater Denver metropolitan area provide past history, complete clinical interviews and psychiatric surveys, and provide biological samples at their baseline visit (29). For this paper, baseline measures were used for participants who provided a home address and a gut microbiome sample. Participants completed clinically administered interviews and self-report survey measures (e.g., demographic characteristics, military history) at their baseline visit.

### Surveys and assessments

Sample characteristics were assessed using the MIRECC Demographics questionnaire, which includes items regarding military service history and standard demographic information. Participants completed the Harvard Food Frequency Questionnaire (Harvard FFQ) 2007 booklet to assess dietary patterns (30). The Harvard FFQ is a validated, 101-item survey that quantifies specific food intake frequencies over the previous 12 months. Veterans also participated in a structured clinical interview, the DSM-5-TR (Diagnostic and Statistical Manual of Mental Disorders, 5th Edition, Text Revision) Axis I Disorders, Research Version, Patient Edition with Psychotic Screen (SCID-5-I/*P* W/PSY SCREEN), a validated method for assessing lifetime diagnoses of psychiatric disorders, and past-month symptoms of PTSD (31). Results were collapsed into broad categories of disorders (e.g., mood disorders, alcohol use disorders, substance use disorders, anxiety disorders, nonaffective psychotic disorders, and “other” DSM-5 diagnoses). From participants’ medical records, we extracted data on veterans’ receipt of benefits, termed “service connection,” related to injuries sustained or conditions developed while serving in the military. This service connection disability rating is represented as a percentage from 0 to 100 and is applied in increments of 10%.

### Gut microbiome sample collection

Fecal samples were collected with double-tipped polyurethane swabs (BD BBL CultureSwab EZ II, Cat No. B220144, Fisher Scientific, Pittsburgh, PA, USA). Participants either collected fecal swabs during an inpatient evaluation at the research facility that were subsequently stored at −80°C or received a prepackaged sampling kit for home use with instructions for sample collection. Fecal swab samples collected at home were mailed back to the research facility, and upon receipt, all the samples were stored at −80°C. For a more in-depth description of sample collection, please refer to Brenner et al. (29).

### Gut microbiome sample processing

Sample DNA was extracted from fecal samples using the PowerSoil DNA Extraction Kit (Cat. No. 12955-4, Qiagen, Valencia, CA, USA) (for further details, see Brenner et al. [29]). Marker genes in isolated DNA were PCR-amplified in duplicate using GoTaq Master Mix (Cat. No. M5133, Promega, Madison, WI, USA) as described in Caporaso et al. (32) using primers 515F–808R. Successful amplification was visualized on an agarose gel prior to pooling. Pooled amplicons were sent to the University of Colorado Anschutz Research Genetic Organization for normalization and sequencing on an Illumina MiSeq.

### Food desert status

Participant residential addresses at the time they participated in the study were geocoded to census tracts via ArcGIS Pro (Version 2.6.0, Esri, Inc.) (33). The USDA Food Access Research Atlas (34) specifies food desert areas by census tract: “Low Access [LA] 1 and 10” or “tracts in which at least 500 people or 33% of the population lives farther than 1 mile (urban) or 10 miles (rural) from the nearest supermarket.” Participants residing in census tract LA 1 and 10 locations were designated into the food desert group, whereas those who fell outside of these locations were designated into the non-food desert group. Participants who reported being unhoused at the time of data collection provided the address for the location at which they were staying that day.

### Data analysis

For dietary quality, we applied the Healthy Eating Index (HEI) to Harvard FFQ data collected for this cohort (35). The HEI-2015 assesses adherence to the USDA’s 2015–2020 U.S. Dietary Guidelines for Americans and is used to evaluate energy-adjusted intake of 13 different dietary groups (e.g., legumes, whole grains, added sugars) and proportional intakes (e.g., density of total protein per 1,000 kcal). HEI analysis yields individual subscales for each variable and an aggregate score ranging from 0 to 100, with 0 being no adherence to dietary guidelines and 100 being perfect adherence.

The HEI was calculated in SAS 9.4 using a macro provided by the National Cancer Institute (36). We examined differences in total HEI and in each dietary subcategory by food desert residence using independent samples *t*-tests or Wilcoxon rank-sum tests, as appropriate. We also examined differences in demographic characteristics, mental health diagnoses, and service connection by food desert residence using independent samples *t*-tests, Wilcoxon rank-sum tests, χ tests, or Fisher’s exact tests, as appropriate. All analyses assumed a two-sided test of hypothesis with a significance level of α = 0.05 and were conducted using SAS 9.4.

Sequencing data were processed using the Quantitative Insights into Microbial Ecology open-source software (QIIME2 v. 2021.8) (37). The Deblur algorithm (38) was used to denoise demultiplexed sequences. SATé-enabled phylogenetic placement (39) analysis was performed to improve the phylogenic tree used in β-diversity measures. Developer-recommended inputs included removal sequences that were not 75% similar to any record in the tree, but no amplicon sequencing variants in the present study met these criteria. Quality-filtered sequences were assigned taxonomic classification based on the silva_128 database (40). Samples were shipped to the facility by 237 participants (69.3% of the samples in the study) with instructions to freeze samples overnight and ship with the provided ice pack overnight. We observed that cold shipping was not consistently used, and/or there were mail issues that prevented samples from remaining cold. Therefore, we used a normalization of the community due to growth approximation and as previously described by Amir et al. (38). Downstream analysis and visualization were performed with the open-source statistical package R v.4.2.1 (41).

For α- and β-diversity and taxonomic evaluations, samples were rarefied to a level of 10,000 sequences per sample. All statistical analyses were adjusted for known covariates that can impact the microbiome, including age (grouped into decades), ethnicity, alcohol use, and gender (42). As the majority of participants who had an alcohol use disorder diagnosis also had a concurrent substance use disorder (78%), we did not specifically adjust for the latter. We also adjusted for housing history, specifically a previous history of homelessness, which we have observed is associated with microbial composition (43).

The α-diversity metrics for observed operational taxonomic units (OTUs), evenness, and Shannon diversity index were calculated on rarefied samples. Each of the α-diversity metrics was skewed, so Tukey’s transformation was conducted in the rcompanion package (44) prior to statistical modeling. Statistical analyses were conducted in R using the “glm” function for generalized linear modeling. β-Diversity was determined using the phyloseq package (45) in R for weighted and unweighted UniFrac distance metrics. β-Diversity was measured using the “adonis2” function of the vegan package (46) after 1,000 permutations. In order to determine the strongest correlates of fecal microbiota composition, we used a stepwise model-building tool for constrained ordination methods based on adjusted *R*^2^, using the UniFrac distance metrics with the “envfit” function of the vegan package. As the majority of participants were male (81.2%), α- and β-diversity measures were calculated with a subset of only males. The observed results altered *P*-values slightly but did not change significance to levels of under or over 0.05, so further stratification of the data was not pursued.

Differential abundance testing was analyzed using the Analysis of Compositions of Microbiomes with Bias Correction (ANCOM-BC) package with the ancombc2 function (47) (grouped by food desert, function of food desert plus five covariates, *P* adjustment with holm, and taxonomic prevalence >25%). A correlation matrix was developed between the top 20 genera and 25 metadata categories through the microbiomeSeq package (48) in R. Correlations were calculated using the Pearson correlation coefficient with Bejamini-Hochberg adjusted *P*-values for repeated measures of metadata categories and food desert status groups. The reporting of fecal microbiome data in this report is consistent with the Strengthening The Organization and Reporting of Microbiome Studies guidelines for human microbiome research (49).

## RESULTS

### Participant characteristics

Demographic characteristics are listed in Table 1. Our final cohort consisted of 342 U.S. veterans, 108 residing in food deserts (32%), and 234 in non-food deserts (68%). The majority of participants identified as male (81.2%) and Caucasian (67.4%), with a mean (± standard deviation) age of 47.9 y (±13.2). Ninety-eight percent of the participants resided in urban areas. Compared to those in the non-food desert group, veterans residing in food deserts were significantly younger (*P* < 0.03). Among participants who identified as being of Hispanic ethnicity, a greater proportion resided in food deserts relative to non-food deserts (*P* < 0.04). There was also a significantly higher percentage of students enrolled in post-secondary education among those residing in food deserts (*P* = 0.02). A significantly greater proportion of veterans living in non-food deserts reported having previously experienced homelessness (*P* < 0.0001) than veterans living in food deserts. In terms of military service history, food desert residents had experienced more deployments (*P* = 0.0006) and more deployments to combat zones (*P* < 0.0002). A significantly higher percentage of veterans living in food deserts had a Veterans Health Administration (VHA) service connection than their non-food desert counterparts (*P* < 0.0002).

**TABLE 1:** Characteristics of the US-VMP cohort, overall and by food desert status

Characteristic	Overall *N* = 342 mean (SD) or *N* (%)	Non-food desert *N* = 23 mean (SD) or *N* (%)	Food desert *N* = 108 mean (SD) or *N* (%)	*P*-value	Statistical test
Age	47.9 (13.2)	49.0 (13.0)	45.6 (13.4)	0.03	Independent samples *t*-test
Sex^*a*^				0.89	Fisher’s exact test
Male	277 (81.2%)	189 (81.2%)	88 (81.5%)		
Female	62 (18.2%)	43 (18.5%)	19 (17.6%)		
Other	2 (0.6%)	1 (0.4%)	1 (0.9%)		
Race^*a*^				0.47	χ^2^
Caucasian/White	227 (67.4%)	154 (66.9%)	73 (68.2%)		
Black or African American	59 (17.5%)	43 (18.7%)	16 (14.9%)		
Other	31 (9.2%)	18 (7.8%)	13 (12.2%)		
Multiracial	20 (5.9%)	15 (6.5%)	5 (4.7%)		
Ethnicity^*a*^				0.04	χ^2^
Hispanic	50 (14.8%)	28 (12.2%)	22 (20.6%)		
Non-Hispanic	287 (85.2%)	202 (87.8%)	85 (79.4%)		
Highest level of education^*a*^				0.02	χ^2^
High school or less	39 (11.4%)	35 (15.0%)	4 (3.7%)		
Some college, no degree	119 (34.9%)	80 (34.3%)	39 (36.1%)		
Associate or bachelor’s degree	132 (38.7%)	87 (37.3%)	45 (41.7%)		
Master’s or doctoral degree	51 (15.0%)	31 (13.3%)	20 (18.5%)		
Marital status^*a*^				<0.0001	χ^2^
Partnered	156 (46.0%)	87 (37.3%)	69 (65.1%)		
Not partnered	183 (54.0%)	146 (62.7%)	37 (34.9%)		
Employment status^*a*^				0.18	χ^2^
Employed full-time	87 (25.8%)	51 (22.7%)	36 (33.6%)		
Employed part-time	37 (11.0%)	27 (11.7%)	10 (9.4%)		
Unemployed, not seeking employment	69 (20.5%)	51 (22.2%)	18 (16.8%)		
Unemployed, seeking employment	52 (15.4%)	39 (17.0%)	13 (12.2%)		
Retired	92 (27.3%)	62 (27.0%)	30 (28.0%)		
Are you currently a student^*a*^?				0.01	χ^2^
Yes	57 (16.7%)	31 (13.3%)	26 (24.1%)		
No	284 (83.3%)	202 (86.7%)	82 (75.9%)		
Are you currently homeless^*a*^?				0.07	χ^2^
No	315 (92.4%)	212 (90.6%)	103 (96.3%)		
Yes	26 (7.6%)	22 (9.4%)	4 (3.7%)		
Episodes of prior homelessness				<0.0001	Wilcoxon rank-sum test
Never experienced homelessness	200 (58.5%)	116 (49.6%)	84 (77.8%)		
Experienced homelessness at least once	142 (41.5%)	118 (50.4%)	24 (22.2%)		
Rurality				0.21	Fisher’s exact test
Urban	335 (97.9%)	231 (98.7%)	104 (96.3%)		
Rural	7 (2.1%)	3 (1.3%)	4 (3.7%)		
Most recent era served^*a*^				0.004	χ^2^
Operation Enduring Freedom/Operation Iraqi Freedom/Operation New Dawn	114 (33.4%)	72 (30.9%)	42 (38.9%)		
Desert Storm	85 (24.9%)	50 (21.5%)	35 (32.4%)		
Other	142 (41.6%)	111 (47.6%)	31 (28.7%)		
Highest military rank				0.01	χ^2^
Enlisted	222 (64.9%)	164 (70.1%)	58 (53.7%)		
Noncommissioned officer	92 (26.9%)	55 (23.5%)	37 (34.2%)		
Officer	28 (8.2%)	15 (6.4%)	13 (12.0%)		
Number of military deployments	2.2 (4.9)	1.6 (2.2)	3.3 (7.9)	0.0006	Wilcoxon rank-sum test
Number of deployments into combat zone	1.1 (2.6)	0.8 (1.3)	1.6 (4.2)	0.0002	Wilcoxon rank-sum test
Military sexual trauma 1^*a*^^*,^c^*^ (MST 1)				0.59	Fisher’s exact test
No	254 (74.7%)	171 (73.7%)	83 (76.9%)		
Yes	86 (25.3%)	61 (26.3%)	25 (23.1%)		
Military sexual trauma 2^*a*^^,^^*d*^				0.72	Fisher’s exact test
No	301 (88.3%)	207 (88.8%)	94 (87.0%)		
Yes	40 (11.7%)	26 (11.2%)	14 (13.0%)		
Branch of military service				0.49	χ^2^
Army	175 (51.2%)	112 (47.9%)	63 (58.3%)		
Air Force	52 (15.2%)	39 (16.7%)	13 (12.0%)		
Navy	60 (17.5%)	41 (17.5%)	19 (17.6%)		
Marine Corps	36 (10.5%)	28 (12.0%)	8 (7.4%)		
Coast Guard	3 (0.9%)	2 (0.85%)	1 (0.9%)		
Multiple branches	16 (4.7%)	12 (5.1%)	4 (3.7%)		
Months of active duty service ^*b*^	82.3 (71.0)	72.6 (66.6)	103.5 (75.8)	<0.0001	Wilcoxon rank-sum test
Months of reserve service ^*b*^	21.3 (48.1)	24.0 (51.1)	15.5 (40.5)	0.03	Wilcoxon rank-sum test
Veteran service connection				0.0002	Fisher’s exact test
0% or not service connected	105 (30.7%)	84 (35.9%)	21 (19.4%)		
10%–20%	23 (6.7%)	21 (9.0%)	2 (1.2%)		
30%–60%	43 (12.6%)	28 (12.0%)	15 (13.9%)		
70%–90%	86 (25.2%)	54 (23.1%)	32 (29.6%)		
100%	85 (24.85%)	47 (20.1%)	38 (35.2%)		

^*a*^ One participant was missing information about sex. Five participants were missing information about their race. Five participants were missing information about ethnicity. One participant was missing information about education. Three participants were missing information about marital status. Five participants were missing information about employment. One participant was missing information about student status. One participant was missing information about current homelessness. One participant was missing information about the most recent era served. Two participants were missing information about MST question 1. One participant was missing information about MST question 2.

^*b*^ One participant was missing information for months served on active duty. Two participants were missing information for months of reserve service.

^*c*^ Question: while you were in the military, did you receive any uninvited and unwanted sexual attention, such as touching, cornering, pressure for sexual favors, or inappropriate verbal remarks?

^*d*^ Question: while you were in the military, did anyone ever use force or the threat of force to have sexual contact with you against your will?

### Dietary outcomes

HEI scores were calculated for food and non-food desert groups and are presented in Table 2. There were no significant differences in dietary quality between groups (*P* = 0.24), with food desert groups having an average HEI score of 66.6 (±10.9) and non-food desert groups having an average HEI of 68.2 (±11.9). Within HEI subcategories, those residing in food deserts reported significantly higher intakes of total fatty acids (*P* < 0.001) than non-food desert participants, but no other statistically significant differences were found in any other HEI subcategories.

**TABLE 2:** Total HEI and component indices, overall and by food desert status

HEI	Overall *N* = 342	Not living in food desert *N* = 234	Living in food desert *N* = 108	*P*-value	Statistical test
	Mean ± SD	Mean ± SD	Mean ± SD		
Total HEI	67.1 ± 11.2	66.6 ± 10.9	68.2 ± 11.9	0.24	Independent samples *t*-test
Total vegetables	4.2 ± 1.2	4.2 ± 1.2	4.2 ± 1.2	0.99	
Beans and greens	4.3 ± 1.3	4.3 ± 1.3	4.3 ± 1.3	0.67	
Total fruits	3.7 ± 1.5	3.6 ± 1.5	4 ± 1.4	0.07	
Whole fruits	4.2 ± 1.4	4.1 ± 1.4	4.3 ± 1.3	0.21	
Total protein foods	3 ± 0.6	3 ± 0.7	2.9 ± 0.6	0.15	
Seafood and plant proteins	4.3 ± 0.8	4.3 ± 0.9	4.4 ± 0.8	0.73	
Whole grains	4.2 ± 2.2	4.3 ± 2.3	4 ± 2.1	0.16	
Dairy	3.2 ± 2.8	3.3 ± 2.9	2.9 ± 2.8	0.19	
Fatty acids	4.5 ± 2.9	4.1 ± 2.8	5.3 ± 3.1	0.001	
Sodium	9 ± 1.6	8.9 ± 1.6	9.2 ± 1.5	0.12	
Saturated fats	5.6 ± 2.8	5.4 ± 2.6	5.8 ± 3.1	0.23	
Added sugars	7 ± 3	7 ± 3.1	7.1 ± 2.8	0.81	
Refined grains	9.9 ± 0.8	9.9 ± 0.7	9.9 ± 1	0.55	Wilcoxon rank-sum test

### Mental health outcomes

Mental health results are presented in Table 3. There was a significantly higher percentage of participants with past-month PTSD symptoms residing in food deserts than non-food desert participants (*P* < 0.04), whereas there were significantly more participants in the non-food desert group diagnosed with substance use disorders (*P* = 0.002) and current alcohol use disorders (*P* = 0.04). No other significant differences were found in mental health outcomes by food desert status.

**TABLE 3:** Prevalence of mental health disorders, overall and by food desert status

DSM-5 disorders identified by SCID-5^*a*^	Overall	Not living in food desert	Living in food desert	*P*-value	
	*N* = 342	*N* = 234	*N* = 108		Statistical test
	*N* (%)	*N* (%)	*N* (%)		
Lifetime mood disorder	184 (54.8%)	123 (53.7%)	61 (57.0%)	0.57	χ^2^
Lifetime nonaffective psychotic disorder	6 (1.8%)	5 (2.2%)	1 (0.9%)	0.67	Fisher’s exact test
Lifetime alcohol use disorder	171 (50.6%)	125 (53.9%)	46 (43.4%)	0.07	χ^2^
Current alcohol use disorder	36 (10.7%)	30 (12.9%)	6 (5.7%)	0.04	χ^2^
Lifetime substance use disorder	113 (33.5%)	93 (40.1%)	20 (19.1%)	0.0002	χ^2^
Anxiety disorder	43 (12.8%)	26 (11.3%)	17 (16.0%)	0.22	χ^2^
Lifetime PTSD	156 (46.4%)	103 (44.6%)	53 (50.4%)	0.32	χ^2^
Current PTSD	85 (25.3%)	51 (22.1%)	34 (32.4%)	0.04	χ^2^
Lifetime other stress disorder	13 (3.9%)	9 (3.9%)	4 (3.8%)	1.0	Fisher’s exact test
Other DSM-5 disorder^*b*^^*c*^	55 (16.5%)	37 (16.2%)	18 (17.1%)	0.82	χ^2^

^*a*^ Six participants were missing information about mood disorders. Two participants were missing information about nonaffective psychotic disorders. Four participants were missing information about alcohol use. Five participants were missing information about substance use. Five participants were missing information about anxiety disorders. Six participants were missing information about PTSD. Five participants were missing information about other stress disorders. Eight participants were missing information about other DSM-5 disorders.

^*b*^ Other lifetime disorders include the following: obsessive-compulsive disorder, hoarding disorder, body dysmorphic disorder, anorexia nervosa, bulimia nervosa, binge eating disorder, avoidant/restrictive food intake disorder, other eating disorders, and other DSM-5 disorders.

^*c*^ Other current disorders include the following: adult attention-deficit/hyperactivity disorder, intermittent explosive disorder, other specified trauma- and stressor-related disorders, anorexia nervosa, bulimia nervosa, binge eating disorder, avoidant/restrictive food intake disorder, other eating disorders, acute stress disorder, hoarding disorder, gambling disorder, insomnia disorder, hypersomnolence disorder, other sleep disorders, body dysmorphic disorder, somatic symptom disorder, illness anxiety disorder, and other DSM-5 disorders.

### Gut microbiome results

α-Diversity did not differ by food desert status in measures of observed OTUs (Wilcoxon test, *P* = 0.96), Shannon diversity index (Wilcoxon test, *P* = 0.67), or Pielou’s evenness (Wilcoxon test, *P* = 0.48) (Fig. S1). Generalized linear modeling with covariates lowered the *P*-values, but not to a significant level, for observed OTUs (glm, *P* = 0.933), Shannon diversity index (glm, *P* = 0.36), and Pielou’s evenness (glm, *P* = 0.335).

**FIG 1:**
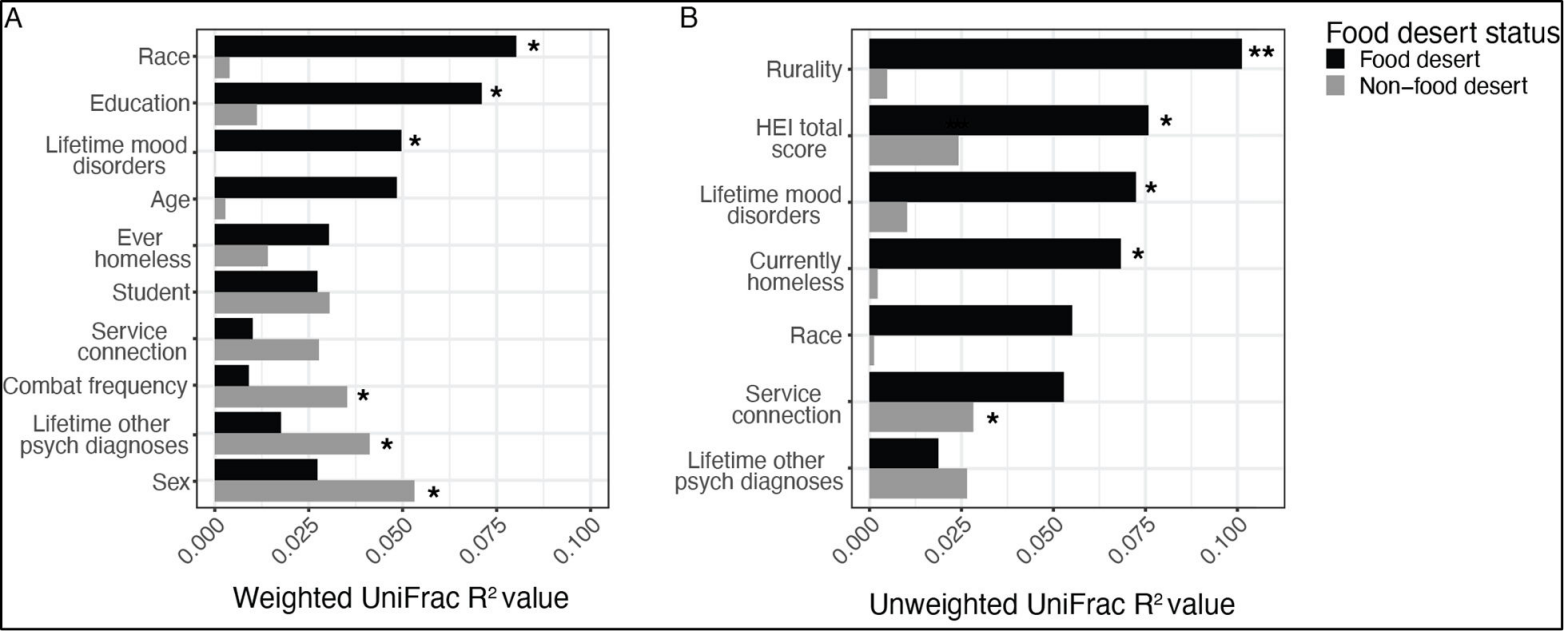
CCA *R*^2^ estimates for β-diversity of the gut microbiome analyzed using (**A**) weighted and (**B**) unweighted UniFrac distance metrics.

For β-diversity, microbial community composition also did not significantly differ for weighted UniFrac distance after adjustment for the five covariates (ADONIS, *P* = 0.651) (Fig. S2a). In contrast, unweighted UniFrac distance, which accounts for phylogenetic differences but not taxonomic relative abundance, was nearly significantly different after adjustment for covariates (ADONIS, *P* = 0.058) (Fig. S2b). To explore correlates of β-diversity further, canonical-correlation analysis (CCA) was performed for weighted and unweighted UniFrac distances. In the CCA using an unweighted UniFrac distance matrix, food desert status was significantly associated with phylogenetic differences, accounting for 2.4% of β-diversity among participants (*R*^2^ = 0.024, *P* = 0.026) (Fig. 1B; Fig. S3). Using the unweighted UniFrac distance metric, categories of rurality, HEI total score, mood disorders, current homelessness, race, and service-connected disabilities all significantly differed between food desert and non-food desert participants. In CCA analysis of the weighted UniFrac distance matrix based on food desert status (Fig. 1A), β-diversity was most explained by statistically significant demographic variables (race [*R*^2^ = 0.08, *P* = 0.02], education level [*R*^2^ = 0.07, *P* = 0.03], sex [*R*^2^ = 0.05, *P* = 0.002]), military-related variables (combat frequency [*R*^2^ = 0.04, *P* = 0.02]), and mental health measures (mood disorder [*R*^2^ = 0.05, *P* = 0.08], other psychiatric diagnosis [*R*^2^ = 0.04, *P* = 0.03]).

The relative abundances of the most prevalent phyla did not differ by food desert status. The most prevalent phyla (mean [*x̅*] relative abundance ± SD) were Firmicutes (49.3% ± 20.9%), Bacteroidetes (37.6% ± 22.5%), Actinobacteria (6.0% ± 8.0%), Proteobacteria (4.8% ± 11.8%), and Verrucomicrobia (1.7% ± 5.0%) (Fig. S4a). At the genus level, the taxa with the highest relative abundances were *Bacteroides* (22.2% ± 18.9%), *Faecalibacterium* (7.7% ± 8.2%), *Blautia* (6.2% ± 7.5%), and *Prevotella* (6.2% ± 11.0%), none of which differed by food desert status (Fig. S4b). No differentially abundant taxa were observed through ANCOM-BC2 analysis with multiple test corrections for genera and covariates. Prior to adjusting for covariates, we observed significant differential abundance for *Barnesiella* (*x̅* = 0.370% food desert, *x̅* = 0.162% non-food desert, *P* = 0.022), *Campylobacter* (*x̅* = 0.064% food desert, *x̅* = 0.185% non-food desert, *P* = 0.007), and *Peptoniphilus* (*x̅* = 0.813% food desert, *x̅* = 0.977% non-food desert, *P* = 0.048).

**FIG 2:**
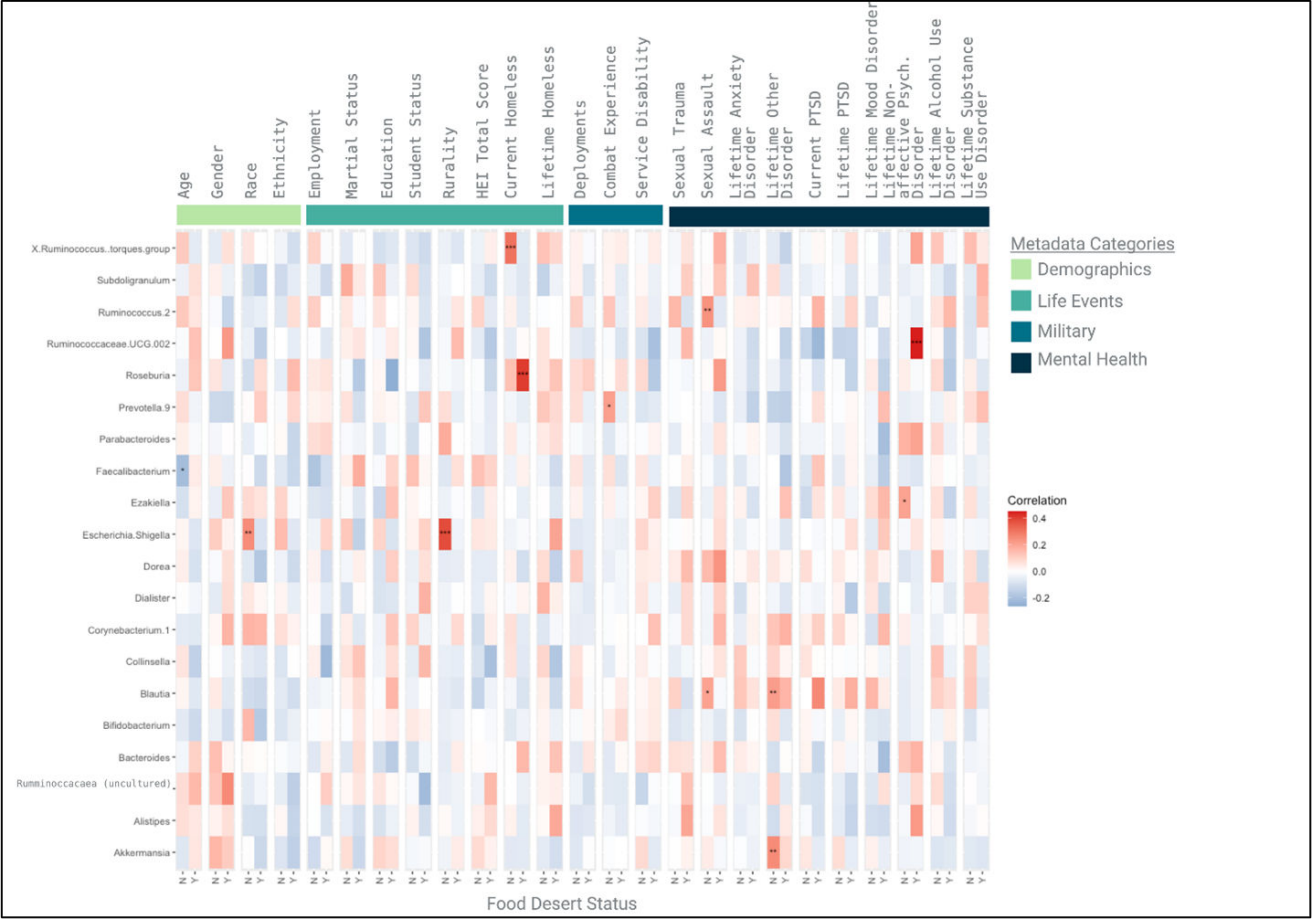
Correlation matrix for most prevalent taxa in relation to metadata, by food desert status, adjusted for multiple comparisons.

Finally, a correlation matrix was developed for the 20 taxa with the highest relative abundances in comparison to 25 participant characteristics, separated by food desert status (Fig. 2). Six correlations were statistically significant and positively associated for non-food desert participants while negatively associated for food desert participants: (i) *Escherichia*-*Shigella* was associated with race and rurality; (ii) *Ruminococcus torques* was associated with current homelessness; (iii) *Ruminococcus 2* and *Blautia* were each associated with a history of military sexual assault; and (iv) *Ezakiella* was associated with nonaffective psychiatric disorder. Lastly, age was negatively correlated with *Faecalibacterium* in non-food desert participants, and *Ruminococcus UCG.002* was positively correlated with nonaffective psychiatric disorder in participants in food deserts.

## DISCUSSION

In this study of food desert status, mental health, and the gut microbiome of military veterans, we explored the complexity inherent in evaluating such distinct yet interrelated factors. The most abundant genera in the present study were similar to those observed in the general population (50). A CCA metric indicated that living in a food desert accounted for 2.4% of β-diversity among veterans. While this finding is statistically significant, it is possible that there are other as-yet unexamined socioeconomic factors related to food that may account for a larger proportion of β-diversity. A factor worthy of future exploration is food insecurity. A financially driven condition of limited or inadequate access to sufficient nutrition (51), food insecurity is associated with an increased risk of adverse health outcomes, including cardiovascular disease, diabetes, and psychiatric illness (52–54). Of note, food insecurity differs from food desert status in that it comprises all of the financial and interpersonal resources that shape access to food, independently or in addition to geographic access. Food insecurity is highly prevalent among veterans in general (55), and the inclusion of measures of food insecurity, which can be more nuanced and granular than a binary food desert status, could yield more informative microbiome-related data. Of note, just recently, Zuniga-Chaves et al. (56) observed that food insecurity mediates the association between low socioeconomic status and decreased α-diversity. Nevertheless, it is also important to note that measuring food insecurity presents its own challenges, related to self-stigma in reporting, particularly among veteran populations (57, 58).

In the context of dietary quality, previous food desert studies have yielded mixed results (59), with some studies reporting differences in nutritious food intake (e.g., fruits and vegetables) (60), and others suggesting that there are no significant differences in dietary habits between food desert and non-food desert residents (61, 62). In our previous assessment of dietary habits among veterans in the US-VMP study (63), we did not observe marked differences in HEI scores. Veterans consumed a largely homogeneous Western-style diet, characterized by high intakes of nutrient-poor refined sugars and processed foods and poor intakes of more nutritious foods (63). Both food desert and non-food desert participants in our study scored higher on the HEI than the national HEI average, which was 57 as of 2018 (36), though the reasons for this are unclear. Total HEI scores did not differ by food desert status, but nevertheless, in the unweighted Unifrac CCA analysis, we observed that total HEI scores accounted for just under 8% of β-diversity among participants living in a food desert. While the reasons for this are also unclear, in addition to geographic proximity to nutritious food, an individual’s subjective perception of how accessible healthy food options are may be an important consideration. The perception as to whether one lives in a food desert and/or a “food swamp” (64) area with a high density of unhealthy food options, which varies by race and ethnicity, has been associated with poorer self-reported dietary quality (65). Definitions of food swamps have yet to be formally operationalized, but such a metric may more accurately reflect participants’ experiences with obtaining the kinds of foods they value to meet their nutritional needs than a measure of geographic proximity to grocery stores.

Race, education, rurality, and mood disorders were significantly positively correlated with residing in a food desert, as were an increased military service connection and current homelessness. Conversely, non-food desert status was significantly correlated with sex, other mental health disorders, and a greater frequency of service in combat. In terms of specific taxa, results were also mixed. For example, *Ruminococcus 2* and *Blautia*, taxa that have been associated with anti-inflammatory functions (66, 67), were both positively correlated with prior history of military sexual assault in non-food desert participants, but not among veterans with a history of sexual assault living in food deserts. Similarly, *Faecalibacterium*, a beneficial genus of bacteria with probiotic properties, was negatively correlated with age. This finding is supported by prior research (68), but in our cohort, there was no such correlation among veterans living in food deserts.

There are several possible explanations for some of our mixed, counterintuitive findings. Some of our observations may have been happenstance, as in the case of the positive correlation between *Escherichia*-*Shigella*, a pathogen, and rurality among non-food desert participants, when only 2% of all participants resided in a rural area. It is also possible that overall socioeconomic status or income are more strongly associated with microbial composition and that factors like food desert status, age, and current student status are simply proxies for participants’ relative burden of social inequity (69, 70). For example, college students, who were significantly more likely to reside in a food desert in this cohort, are inherently at a stage of life with diminished earning potential and at a financial disadvantage compared to their older counterparts (71). Participants with a prior history of experiencing homelessness were also more likely to reside in a food desert. There is evidence to suggest that a history of homelessness is correlated with reduced long-term earning potential (72, 73), once again suggesting that food desert status may have been a proxy for income.

Other demographic factors such as race and ethnicity were associated with food desert status, and race in particular accounted for 7% to 10% of β-diversity, depending on the β-diversity metric. While there is evidence for racial differences in gut microbial compositions and related health outcomes (74, 75), there is also strong evidence that these variations may be due to socioeconomic inequities rather than biological differences (27, 76).

We also observed that, as in previous research (77), mental health is significantly associated with microbial composition. While we observed differences in correlations by food desert status (mood disorders among food desert residents, other psychiatric diagnoses among non-food desert residents), we cannot determine that the disorder in question is truly driving the association between microbial composition and food desert status. Psychiatric conditions, whether a mood disorder, a schizoaffective condition, or PTSD, can all exert a profound influence on how people experience social inequities (78), and differentiating by food desert status did not allow for parsing out how precisely these conditions manifest or how they shape veterans’ behavior as they experience their environments.

Finally, in taking a broader view of our findings, we suggest that military veterans’ unique exposures and characteristics constitute a population that still merits more detailed investigation in microbiome research. In this cohort, greater frequency of service in combat, increased military service connection, PTSD symptoms, and a history of military sexual assault were all to some degree associated with β-diversity among veterans residing in a food desert. The body of literature on gut microbiota in military veterans remains too small to draw firm conclusions regarding factors that may be driving these correlations. Nevertheless, our findings suggest that while veterans have much in common with the general population in terms of microbial associations, general demographics, and common mental health disorders, their service-related exposures may exert a unique influence on their subsequent socioeconomic and environmental characteristics, which may in turn shape microbial composition.

Therefore, we propose that future microbiome studies expand on our findings not only by examining more veteran populations but also by evaluating populations that have had exposures comparable to those of veterans, namely, regimented-style living combined with profound physical and psychological stressors. For example, studies of people in communal living settings, such as barrack-style emergency housing, residential rehabilitation programs for youth, and cloistered religious communities, and who have experienced stressful events, such as political unrest or violence, crime, war, or natural disasters, may provide insights into how such exposures shape subsequent mental health-related and socioeconomic correlations with the microbiome.

It is also important to note that only a small percentage of the United States population serves in the military, with socioeconomic factors playing an important role in terms of individuals enlisting. To increase ecological validity, microbiome studies investigating the influence of factors over time may benefit from the use of an approach that Dowd and Renson (79) termed “social epidemiology of the microbiome.” Dowd and Renson’s approach not only encompasses all of the environmental and behavioral exposures that can shape microbiota at the time of data sampling but also evaluates the exposures and stressors across a person’s life course, all in a holistic socioeconomic, psychological, and cultural context. In terms of veterans, this would allow for increased understanding regarding whether or not pre (e.g., adverse childhood experiences), military (e.g., combat), and post-military (e.g., homelessness) exposures may influence microbial composition over time.

### Limitations

This study has a number of limitations. The binary categorization of food desert status may have significant limitations, with recent studies moving toward more nuanced descriptions of areas of food landscapes, including machine learning-based identification of food desert status (80). Of note, food swamps have been shown to be a better predictor of adverse health outcomes (e.g., obesity) than food deserts (81), but current USDA map data, which were our primary source, do not provide more than a binary differentiation. Moreover, whether one resides in a food desert or not does not necessarily indicate one’s actual food choices, and many residents may not be aware that they reside in a food desert. Next, 26 participants reported being unhoused (no permanent address) at the time of data collection, and while they provided the addresses of the locations at which they were residing on the given day, this may have been an inaccurate reflection of their long-term food desert status. Although we collected data on service connections, we were not able to collect income data on the participants in this cohort, limiting our ability to parse out specific financial influences on food desert associations. Additional measures, such as income, income sources (e.g., employment, Social Security), transportation access, subjective food security, and purchasing locations for food (i.e., near home versus near work), are all likely influential factors whose absence in this study may limit the validity of our findings. It is also worth noting that food frequency questionnaires and the HEI are limited in their ability to accurately assess dietary patterns, and more accurate measures, such as multiple 24-h recalls (82), should be used in future studies, though dietary patterns among our participants were overwhelmingly homogeneous. We were not able to assess species-level associations, and the genus- and phylum-level correlations did not allow us to identify if food desert status was associated with a truly “beneficial” taxon. Our sampling method, like other large-scale fecal studies, directed individuals to ship the samples after freezing, a request that was not always adhered to in practice. Lastly, as data from only one time point were analyzed, if and how associations between food desert status, related covariates, and the gut microbiome may fluctuate over time could not be evaluated.

### Conclusion

Overall, our findings suggest that food desert status had a modest association with gut microbial composition, though other variables, such as race, education, and mood disorders, were more robustly associated with this outcome of interest. In addition, veterans residing in a food desert differed from their non-food desert counterparts in demographic characteristics, combat history while in the military, and mental health diagnoses, but not in dietary quality. Moreover, while food desert status and related covariates accounted for some variance in β-diversity, it is likely that our use of a binary food desert metric was insufficiently robust to account for the complex environmental, psychosocial, and economic forces that shape veterans’ health and functioning. We propose, therefore, that future research should use a social epidemiological approach to characterizing the life course of factors that impact the gut microbiome in this unique population.

## Data Availability

Demultiplexed sequences are deposited in the NCBI Sequence Read Archive (BioProject accession ID: PRJNA1010779).

## SUPPLEMENTAL MATERIAL

Supplemental Figures msystems.00717-23-s0001.pdf Fig. S1 to S4.

Supplemental Legends msystems.00717-23-s0002.docx Legends for supplemental figures.

ASM does not own the copyrights to Supplemental Material that may be linked to, or accessed through, an article. The authors have granted ASM a non-exclusive, world-wide license to publish the Supplemental Material files. Please contact the corresponding author directly for reuse.

## References

[1] Allen J, Balfour R, Bell R, Marmot M. 2014. Social determinants of mental health. Int Rev Psychiatry 26:392–407. doi:10.3109/09540261.2014.928270 25137105

[2] Agid O, Kohn Y, Lerer B. 2000. Environmental stress and psychiatric illness. Biomed Pharmacother 54:135–141. doi:10.1016/S0753-3322(00)89046-0 10840590

[3] Choi T-Y, Choi YP, Koo JW. 2020. Mental disorders linked to crosstalk between the gut microbiome and the brain. Exp Neurobiol 29:403–416. doi:10.5607/en20047 33139585 PMC7788310

[4] Peirce JM, Alviña K. 2019. The role of inflammation and the gut microbiome in depression and anxiety. J Neurosci Res 97:1223–1241. doi:10.1002/jnr.24476 31144383

[5] USDA ERS - Definitions of Food Security. n.d. Available from. Available from: https://www.ers.usda.gov/topics/food-nutrition-assistance/food-security-in-the-us/definitions-of-food-security

[6] Alviola PA, Nayga RM, Thomsen MR, Wang Z. 2013. Determinants of food deserts. American J Agri Economics 95:1259–1265. doi:10.1093/ajae/aat029

[7] Sadler RC, Gilliland JA, Arku G. 2016. Theoretical issues in the ‘food desert’ debate and ways forward. GeoJournal 81:443–455. doi:10.1007/s10708-015-9634-6

[8] Kelli HM, Hammadah M, Ahmed H, Ko Y-A, Topel M, Samman-Tahhan A. 2017. Association between living in food deserts and cardiovascular risk. Circ Cardiovasc Qual Outcomes 10:e003532. doi:10.1161/CIRCOUTCOMES.116.003532 28904075 PMC5810926

[9] Morris AA, McAllister P, Grant A, Geng S, Kelli HM, Kalogeropoulos A, Quyyumi A, Butler J. 2019. Relation of living in a “food desert” to recurrent hospitalizations in patients with heart failure. Am J Cardiol 123:291–296. doi:10.1016/j.amjcard.2018.10.004 30442360 PMC6497163

[10] Kelli HM, Kim JH, Samman Tahhan A, Liu C, Ko Y-A, Hammadah M. 2019. Living in food deserts and adverse cardiovascular outcomes in patients with cardiovascular disease. J Am Heart Assoc 8:e010694. doi:10.1161/JAHA.118.010694 30741595 PMC6405658

[11] Bremner JD, Moazzami K, Wittbrodt MT, Nye JA, Lima BB, Gillespie CF, Rapaport MH, Pearce BD, Shah AJ, Vaccarino V. 2020. Diet, stress and mental health. Nutrients 12:2428. doi:10.3390/nu12082428 32823562 PMC7468813

[12] Earnshaw VA, Karpyn A. 2020. Understanding stigma and food inequity: a conceptual framework to inform research, intervention, and policy. Transl Behav Med 10:1350–1357. doi:10.1093/tbm/ibaa087 33421077 PMC8218858

[13] Godos J, Currenti W, Angelino D, Mena P, Castellano S, Caraci F, Galvano F, Del Rio D, Ferri R, Grosso G. 2020. Diet and mental health: review of the recent updates on molecular mechanisms. Antioxidants (Basel) 9:346. doi:10.3390/antiox9040346 32340112 PMC7222344

[14] Liu RT, Walsh RFL, Sheehan AE. 2019. Prebiotics and probiotics for depression and anxiety: a systematic review and meta-analysis of controlled clinical trials. Neurosci Biobehav Rev 102:13–23. doi:10.1016/j.neubiorev.2019.03.023 31004628 PMC6584030

[15] Loughman A, Staudacher HM, Rocks T, Ruusunen A, Marx W, O’Neil A. 2021. Diet and mental health. Microbes and the Mind 32:100–112. doi:10.1159/isbn.978-3-318-06856-6 34032648

[16] Compton MT, Ku BS. 2023. Prevalence of food insecurity and living in a food desert among individuals with serious mental illnesses in public mental health clinics. Community Ment Health J 59:357–362. doi:10.1007/s10597-022-01013-w 35963919 PMC10209833

[17] Crowe J, Lacy C, Columbus Y. 2018. Barriers to food security and community stress in an urban food desert. Urban Science 2:46. doi:10.3390/urbansci2020046

[18] Walker RE, Fryer CS, Butler J, Keane CR, Kriska A, Burke JG. 2011. Factors influencing food buying practices in residents of a low-income food desert and a low-income food oasis. Journal of Mixed Methods Research 5:247–267. doi:10.1177/1558689811412971

[19] ERS. 2021. Economic Research Service (ERS) Usdoau. USDA ERS - key Statistics & Graphics, . In

[20] Substance Abuse and Mental Health Services Administration. 2022. Key Substance Use and Mental Health Indicators in the United States: Results from the 2021 National Survey on Drug Use and Health (HHS Publication No.PEP22-07-01-005, NSDUH Series H-57). Center for Behavioral Health Statistics and Quality, Substance Abuse and Mental Health Services Administration Rockville, MD. Available from: https://www.samhsa.gov/data/report/2021-nsduh-annual-national-report

[21] Trivedi RB, Post EP, Sun H, Pomerantz A, Saxon AJ, Piette JD, Maynard C, Arnow B, Curtis I, Fihn SD, Nelson K. 2015. Prevalence, comorbidity, and prognosis of mental health among US Veterans. Am J Public Health 105:2564–2569. doi:10.2105/AJPH.2015.302836 26474009 PMC4638236

[22] Fulton JJ, Calhoun PS, Wagner HR, Schry AR, Hair LP, Feeling N, Elbogen E, Beckham JC. 2015. The prevalence of posttraumatic stress disorder in operation enduring freedom/operation Iraqi freedom (OEF/OIF) Veterans: a meta-analysis. J Anxiety Disord 31:98–107. doi:10.1016/j.janxdis.2015.02.003 25768399

[23] Muirhead L, Echt KV, Alexis AM, Mirk A. 2022. Social determinants of health: considerations for care of older Veterans. Nurs Clin North Am 57:329–345. doi:10.1016/j.cnur.2022.04.002 35985723

[24] Pugh MJ, Swan AA, Carlson KF, Jaramillo CA, Eapen BC, Dillahunt-Aspillaga C, Amuan ME, Delgado RE, McConnell K, Finley EP, Grafman JH, Trajectories of Resilience and Complex Comorbidity Study Team. 2018. Traumatic brain injury severity, comorbidity, social support, family functioning, and community reintegration among Veterans of the Afghanistan and Iraq wars. Arch Phys Med Rehabil 99:S40–S49. doi:10.1016/j.apmr.2017.05.021 28648681

[25] Kleykamp M. 2013. Unemployment, earnings and enrollment among post 9/11 Veterans. Soc Sci Res 42:836–851. doi:10.1016/j.ssresearch.2012.12.017 23521998

[26] Ishaq SL, Parada FJ, Wolf PG, Bonilla CY, Carney MA, Benezra A, Wissel E, Friedman M, DeAngelis KM, Robinson JM, Fahimipour AK, Manus MB, Grieneisen L, Dietz LG, Pathak A, Chauhan A, Kuthyar S, Stewart JD, Dasari MR, Nonnamaker E, Choudoir M, Horve PF, Zimmerman NB, Kozik AJ, Darling KW, Romero-Olivares AL, Hariharan J, Farmer N, Maki KA, Collier JL, O’Doherty KC, Letourneau J, Kline J, Moses PL, Morar N. 2021. Introducing the microbes and social equity working group: considering the microbial components of social, environmental, and health justice. mSystems 6:e0047121. doi:10.1128/mSystems.00471-21 34313460 PMC8407420

[27] Amato KR, Arrieta M-C, Azad MB, Bailey MT, Broussard JL, Bruggeling CE, Claud EC, Costello EK, Davenport ER, Dutilh BE, Swain Ewald HA, Ewald P, Hanlon EC, Julion W, Keshavarzian A, Maurice CF, Miller GE, Preidis GA, Segurel L, Singer B, Subramanian S, Zhao L, Kuzawa CW. 2021. The human gut microbiome and health inequities. Proc Natl Acad Sci U S A 118:e2017947118. doi:10.1073/pnas.2017947118 34161260 PMC8237592

[28] Rook GAW. 2022. Evolution, the immune system, and the health consequences of socioeconomic inequality. mSystems 7:e0143821. doi:10.1128/msystems.01438-21 35285679 PMC9040728

[29] Brenner LA, Hoisington AJ, Stearns-Yoder KA, Stamper CE, Heinze JD, Postolache TT, Hadidi DA, Hoffmire CA, Stanislawski MA, Lowry CA. 2018. Military-related exposures, social determinants of health, and dysbiosis: the United States-Veteran microbiome project (US-VMP). Front Cell Infect Microbiol 8:400. doi:10.3389/fcimb.2018.00400 30510919 PMC6252388

[30] Willett WC, Reynolds RD, Cottrell-Hoehner S, Sampson L, Browne ML. 1987. Validation of a semi-quantitative food frequency questionnaire: comparison with a 1-year diet record. J Am Diet Assoc 87:43–47. 3794132

[31] American psychiatric association. 2013. Diagnostic and statistical Manual of mental disorders. 5th Edn. American psychiatric association, Arlington, VA. doi:10.1176/appi.books.9780890425596

[32] Caporaso JG, Lauber CL, Walters WA, Berg-Lyons D, Huntley J, Fierer N, Owens SM, Betley J, Fraser L, Bauer M, Gormley N, Gilbert JA, Smith G, Knight R. 2012. Ultra-high-throughput microbial community analysis on the illumina HiSeq and MiSeq platforms. ISME J 6:1621–1624. doi:10.1038/ismej.2012.8 22402401 PMC3400413

[33] Awaludin N. 2010. Geographical information systems with ArcGIS 9. X principles, techniques, applications and management. Penerbit Andi.

[34] ERS. 2022. Food access research Atlas, . In U.S. Department of Agriculture (USDA)

[35] Krebs-Smith SM, Pannucci TE, Subar AF, Kirkpatrick SI, Lerman JL, Tooze JA, Wilson MM, Reedy J. 2018. Update of the healthy eating index: HEI-2015. J Acad Nutr Diet 118:1591–1602. doi:10.1016/j.jand.2018.05.021 30146071 PMC6719291

[36] 2021. The healthy eating index – population ratio method. National Cancer Institute.

[37] Bolyen E, Rideout JR, Dillon MR, Bokulich NA, Abnet CC, Al-Ghalith GA, Alexander H, Alm EJ, Arumugam M, Asnicar F, Bai Y, Bisanz JE, Bittinger K, Brejnrod A, Brislawn CJ, Brown CT, Callahan BJ, Caraballo-Rodríguez AM, Chase J, Cope EK, Da Silva R, Diener C, Dorrestein PC, Douglas GM, Durall DM, Duvallet C, Edwardson CF, Ernst M, Estaki M, Fouquier J, Gauglitz JM, Gibbons SM, Gibson DL, Gonzalez A, Gorlick K, Guo J, Hillmann B, Holmes S, Holste H, Huttenhower C, Huttley GA, Janssen S, Jarmusch AK, Jiang L, Kaehler BD, Kang KB, Keefe CR, Keim P, Kelley ST, Knights D, Koester I, Kosciolek T, Kreps J, Langille MGI, Lee J, Ley R, Liu Y-X, Loftfield E, Lozupone C, Maher M, Marotz C, Martin BD, McDonald D, McIver LJ, Melnik AV, Metcalf JL, Morgan SC, Morton JT, Naimey AT, Navas-Molina JA, Nothias LF, Orchanian SB, Pearson T, Peoples SL, Petras D, Preuss ML, Pruesse E, Rasmussen LB, Rivers A, Robeson MS, Rosenthal P, Segata N, Shaffer M, Shiffer A, Sinha R, Song SJ, Spear JR, Swafford AD, Thompson LR, Torres PJ, Trinh P, Tripathi A, Turnbaugh PJ, Ul-Hasan S, van der Hooft JJJ, Vargas F, Vázquez-Baeza Y, Vogtmann E, von Hippel M, Walters W, Wan Y, Wang M, Warren J, Weber KC, Williamson CHD, Willis AD, Xu ZZ, Zaneveld JR, Zhang Y, Zhu Q, Knight R, Caporaso JG. 2019. Reproducible, interactive, scalable and extensible microbiome data science using QIIME 2. Nat Biotechnol 37:852–857. doi:10.1038/s41587-019-0252-6 31341288 PMC7015180

[38] Amir A, McDonald D, Navas-Molina JA, Kopylova E, Morton JT, Zech Xu Z, Kightley EP, Thompson LR, Hyde ER, Gonzalez A, Knight R. 2017. Deblur rapidly resolves single-nucleotide community sequence patterns. mSystems 2:e00191-16. doi:10.1128/mSystems.00191-16 28289731 PMC5340863

[39] Janssen S, McDonald D, Gonzalez A, Navas-Molina JA, Jiang L, Xu ZZ, Winker K, Kado DM, Orwoll E, Manary M, Mirarab S, Knight R. 2018. Phylogenetic placement of exact amplicon sequences improves associations with clinical information. mSystems 3:e00021-18. doi:10.1128/mSystems.00021-18 29719869 PMC5904434

[40] Quast C, Pruesse E, Yilmaz P, Gerken J, Schweer T, Yarza P, Peplies J, Glöckner FO. 2013. The SILVA ribosomal RNA gene database project: improved data processing and web-based tools. Nucleic Acids Res 41:D590–6. doi:10.1093/nar/gks1219 23193283 PMC3531112

[41] Team RC. 2013. R: a language and environment for statistical computing. R foundation for statistical computing, Vienna, Austria. Available from: http://www.R-project org/

[42] Vujkovic-Cvijin I, Sklar J, Jiang L, Natarajan L, Knight R, Belkaid Y. 2020. Host variables confound gut microbiota studies of human disease. Nature 587:448–454. doi:10.1038/s41586-020-2881-9 33149306 PMC7677204

[43] Hoisingt A-Y K, Stamper C, Holiday R, Brostow D, Penzenik M, Forster J, Postolache T, Lowry C, Brenner L. 2023. Association of homelessness and diet on the gut microbiome: a United States-Veteran microbiome project (US-VMP) study. 10.1128/msystems.01021-23 PMC10804991 38132705

[44] rcompanion MSS. 2023. Functions to support extension education program evaluation. version 2.4.30 ed. Rutgers cooperative extension, New Brunswick, New Jersey.

[45] McMurdie PJ, Holmes S. 2013. Phyloseq: an R package for reproducible interactive analysis and graphics of microbiome census data. PLoS One 8:e61217. doi:10.1371/journal.pone.0061217 23630581 PMC3632530

[46] Bermejo A, Pardo JL, Morales J, Cano A. 2016. Comparative study of bioactive components and quality from juices of different mandarins: discriminant multivariate analysis of their primary and secondary metabolites. AS 07:341–351. doi:10.4236/as.2016.76035

[47] Lin H, Peddada SD. 2020. Analysis of compositions of microbiomes with bias correction. Nat Commun 11:3514. doi:10.1038/s41467-020-17041-7 32665548 PMC7360769

[48] Ssekagiri A, Sloan W, editors. “microbiomeSeq: an R package for analysis of microbial communities in an environmental context” ISCB Africa ASBCB Conference; ISCB Kumasi, 2017

[49] Mirzayi C, Renson A, Genomic Standards Consortium, Massive Analysis and Quality Control Society, Zohra F, Elsafoury S, Geistlinger L, Kasselman LJ, Eckenrode K, van de Wijgert J, Loughman A, Marques FZ, MacIntyre DA, Arumugam M, Azhar R, Beghini F, Bergstrom K, Bhatt A, Bisanz JE, Braun J, Bravo HC, Buck GA, Bushman F, Casero D, Clarke G, Collado MC, Cotter PD, Cryan JF, Demmer RT, Devkota S, Elinav E, Escobar JS, Fettweis J, Finn RD, Fodor AA, Forslund S, Franke A, Furlanello C, Gilbert J, Grice E, Haibe-Kains B, Handley S, Herd P, Holmes S, Jacobs JP, Karstens L, Knight R, Knights D, Koren O, Kwon DS, Langille M, Lindsay B, McGovern D, McHardy AC, McWeeney S, Mueller NT, Nezi L, Olm M, Palm N, Pasolli E, Raes J, Redinbo MR, Rühlemann M, Balfour Sartor R, Schloss PD, Schriml L, Segal E, Shardell M, Sharpton T, Smirnova E, Sokol H, Sonnenburg JL, Srinivasan S, Thingholm LB, Turnbaugh PJ, Upadhyay V, Walls RL, Wilmes P, Yamada T, Zeller G, Zhang M, Zhao N, Zhao L, Bao W, Culhane A, Devanarayan V, Dopazo J, Fan X, Fischer M, Jones W, Kusko R, Mason CE, Mercer TR, Sansone S-A, Scherer A, Shi L, Thakkar S, Tong W, Wolfinger R, Hunter C, Segata N, Huttenhower C, Dowd JB, Jones HE, Waldron L. 2021. Reporting guidelines for human microbiome research: the STORMS checklist. Nat Med 27:1885–1892. doi:10.1038/s41591-021-01552-x 34789871 PMC9105086

[50] Guilloux C-A, Lamoureux C, Beauruelle C, Héry-Arnaud G. 2021. Porphyromonas: a neglected potential key genus in human microbiomes. Anaerobe 68:102230. doi:10.1016/j.anaerobe.2020.102230 32615270

[51] Cypel YS, Katon JG, Schure MB, Smith S. 2020. Food insecurity in US military Veterans. Food Nutr Bull 41:399–423. doi:10.1177/0379572120963952 33356537

[52] Gundersen C, Ziliak JP. 2015. Food insecurity and health outcomes. Health Affairs 34:1830–1839. doi:10.1377/hlthaff.2015.0645 26526240

[53] Mendy VL, Vargas R, Cannon-Smith G, Payton M, Enkhmaa B, Zhang L. 2018. Food insecurity and cardiovascular disease risk factors among mississippi adults. Int J Environ Res Public Health 15:2016. doi:10.3390/ijerph15092016 30223555 PMC6165024

[54] Pourmotabbed A, Moradi S, Babaei A, Ghavami A, Mohammadi H, Jalili C, Symonds ME, Miraghajani M. 2020. Food insecurity and mental health: a systematic review and meta-analysis. Public Health Nutr 23:1778–1790. doi:10.1017/S1368980020001512 32174292 PMC10200655

[55] Pooler JA, Srinivasan M, Miller Z, Mian P. 2021. Prevalence and risk factors for food insecurity among low-income US military Veterans. Public Health Rep 136:618–625. doi:10.1177/0033354920974662 33478378 PMC8361571

[56] Zuniga-Chaves I, Eggers S, Kates AE, Safdar N, Suen G, Malecki KMC. 2023. Neighborhood socioeconomic status is associated with low diversity gut microbiomes and multi-drug resistant microorganism colonization. NPJ Biofilms Microbiomes 9:61. doi:10.1038/s41522-023-00430-3 37640705 PMC10462741

[57] Cohen AJ, Dosa DM, Rudolph JL, Halladay CW, Heisler M, Thomas KS. 2022. Risk factors for veteran food insecurity: findings from a national US department of veterans affairs food insecurity screener. Public Health Nutr 25:819–828. doi:10.1017/S1368980021004584 34743780 PMC8957505

[58] Wax SG, Stankorb SM. 2016. Prevalence of food insecurity among military households with children 5 years of age and younger. Public Health Nutr 19:2458–2466. doi:10.1017/S1368980016000422 26976798 PMC10271166

[59] Caspi CE, Sorensen G, Subramanian SV, Kawachi I. 2012. The local food environment and diet: a systematic review. Health Place 18:1172–1187. doi:10.1016/j.healthplace.2012.05.006 22717379 PMC3684395

[60] Rose D, Richards R. 2004. Food store access and household fruit and vegetable use among participants in the US food stamp program. Public Health Nutr 7:1081–1088. doi:10.1079/PHN2004648 15548347

[61] Pearson T, Russell J, Campbell MJ, Barker ME. 2005. “Do 'food deserts' influence fruit and vegetable consumption?--a cross-sectional study”. Appetite 45:195–197. doi:10.1016/j.appet.2005.04.003 15927303

[62] Woodruff RC, Haardörfer R, Raskind IG, Hermstad A, Kegler MC. 2020. Comparing food desert residents with non-food desert residents on grocery shopping behaviours, diet and BMI: results from a propensity score analysis. Public Health Nutr 23:806–811. doi:10.1017/S136898001900363X 31957629 PMC8924878

[63] Brostow DP, Stamper CE, Stanislawski MA, Stearns-Yoder KA, Schneider A, Postolache TT, Forster JE, Hoisington AJ, Lowry CA, Brenner LA. 2021. Dietary habits and the gut microbiota in military veterans: results from the United States-veteran microbiome project (US-VMP). Gut. Microb 2. doi:10.1017/gmb.2021.1 PMC11406408 39296320

[64] Osorio AE, Corradini MG, Williams JD. 2013. Remediating food deserts, food swamps, and food brownfields: helping the poor access nutritious, safe, and affordable food. AMS Rev 3:217–231. doi:10.1007/s13162-013-0049-6

[65] Cooksey Stowers K, Jiang Q, Atoloye AT, Lucan S, Gans K. 2020. Racial differences in perceived food swamp and food desert exposure and disparities in self-reported dietary habits. Int J Environ Res Public Health 17:7143. doi:10.3390/ijerph17197143 33003573 PMC7579470

[66] La Reau AJ, Suen G. 2018. The Ruminococci: key symbionts of the gut ecosystem. J Microbiol 56:199–208. doi:10.1007/s12275-018-8024-4 29492877

[67] Liu X, Mao B, Gu J, Wu J, Cui S, Wang G, Zhao J, Zhang H, Chen W. 2021. Blautia-a new functional genus with potential probiotic properties? Gut Microbes 13:1–21. doi:10.1080/19490976.2021.1875796 PMC7872077 33525961

[68] De Filippis F, Pasolli E, Ercolini D. 2020. Newly explored Faecalibacterium diversity is connected to age, lifestyle, geography, and disease. Curr Biol 30:4932–4943. doi:10.1016/j.cub.2020.09.063 33065016

[69] Kolenikov S, Angeles G. 2009. Socioeconomic status measurement with discrete proxy variables: is principal component analysis a reliable answer. Rev Income Wealth 55:128–165. doi:10.1111/j.1475-4991.2008.00309.x

[70] Moss JL, Johnson NJ, Yu M, Altekruse SF, Cronin KA. 2021. Comparisons of individual- and area-level socioeconomic status as proxies for individual-level measures: evidence from the mortality disparities in American communities study. Popul Health Metr 19:1. doi:10.1186/s12963-020-00244-x 33413469 PMC7792135

[71] Wilmoth JM, London AS, Heflin CM. 2015. Economic well-being among older-adult households: variation by veteran and disability status. J Gerontol Soc Work 58:399–419. doi:10.1080/01634372.2015.1019657 25750998 PMC4509619

[72] Metraux S, Fargo JD, Eng N, Culhane DP. 2018. Employment and earnings trajectories during two decades among adults in New York city homeless shelters. Cityscape 20:173–202.

[73] Rosenberg R, Kim Y. 2018. Aging out of foster care: homelessness, post-secondary education, and employment. Journal of Public Child Welfare 12:99–115. doi:10.1080/15548732.2017.1347551

[74] Farhana L, Antaki F, Murshed F, Mahmud H, Judd SL, Nangia-Makker P, Levi E, Yu Y, Majumdar AP. 2018. Gut microbiome profiling and colorectal cancer in African Americans and Caucasian Americans. World J Gastrointest Pathophysiol 9:47–58. doi:10.4291/wjgp.v9.i2.47 30283710 PMC6163128

[75] Wilson AS, Koller KR, Ramaboli MC, Nesengani LT, Ocvirk S, Chen C, Flanagan CA, Sapp FR, Merritt ZT, Bhatti F, Thomas TK, O’Keefe SJD. 2020. Diet and the human gut microbiome: an international review. Dig Dis Sci 65:723–740. doi:10.1007/s10620-020-06112-w 32060812 PMC7117800

[76] Nieves Delgado A, Baedke J. 2021. Does the human microbiome tell us something about race. Humanit Soc Sci Commun 8:1–12. doi:10.1057/s41599-021-00772-3

[77] Du Toit A. 2019. The gut microbiome and mental health. Nat Rev Microbiol 17:196–196. doi:10.1038/s41579-019-0163-z 30760901

[78] Smith HJ, Huo YJ. 2014. Relative deprivation: how subjective experiences of inequality influence social behavior and health. Policy Insights Behav Brain Sci 1:231–238. doi:10.1177/2372732214550165

[79] Dowd JB, Renson A. 2018. "Under the skin" and into the gut: social epidemiology of the microbiome. Curr Epidemiol Rep 5:432–441. doi:10.1007/s40471-018-0167-7 30596004 PMC6290701

[80] Zhao YD. 2020. Doctoral thesis: machine learning based identification of food desert effect in Urbun development. Cleveland State University, Cleveland, OH, USA.

[81] Cooksey-Stowers K, Schwartz MB, Brownell KD. 2017. Food swamps predict obesity rates better than food deserts in the United States. Int J Environ Res Public Health 14:11–1366. doi:10.3390/ijerph14111366 PMC5708005 29135909

[82] Burrows TL, Ho YY, Rollo ME, Collins CE. 2019. Validity of dietary assessment methods when compared to the method of doubly labeled water: a systematic review in adults. Front Endocrinol (Lausanne) 10:850. doi:10.3389/fendo.2019.00850 31920966 PMC6928130

