# Supplementary material for: Food desert residence has limited impact on veteran fecal microbiome composition: a U.S. Veteran Microbiome Project study
Source: mSystems. 2023 Oct 24;8(6):e00717-23. doi: 10.1128/msystems.00717-23 (PMC10734509; doi:10.1128/msystems.00717-23)
Supplement: Supplemental Figures — Fig. S1 to S4. [file msystems.00717-23-s0001.pdf]

Supplemental Figure 1

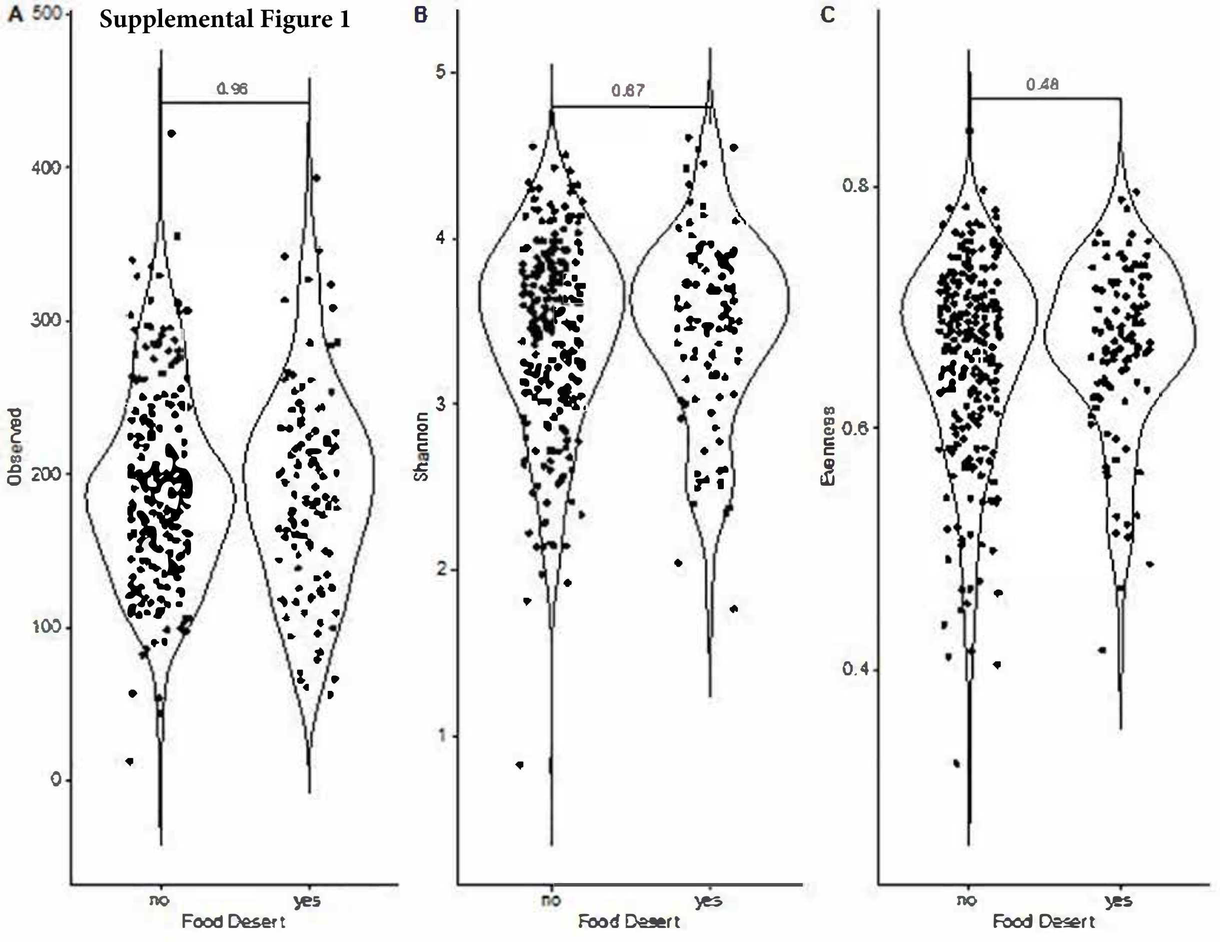

Supplemental Figure 2

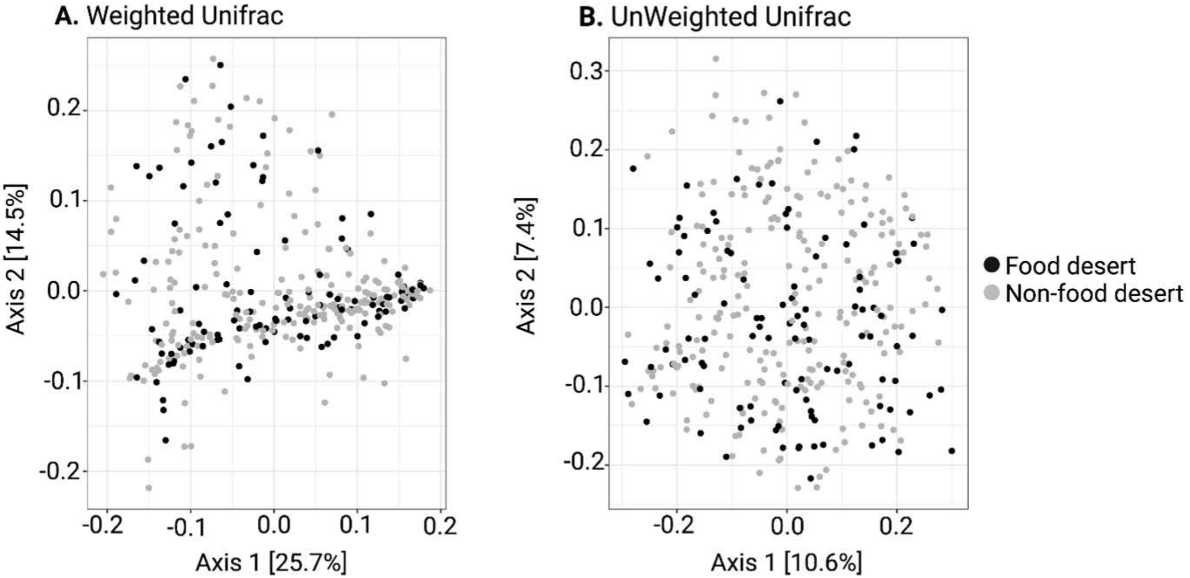

Supplemental Figure 3

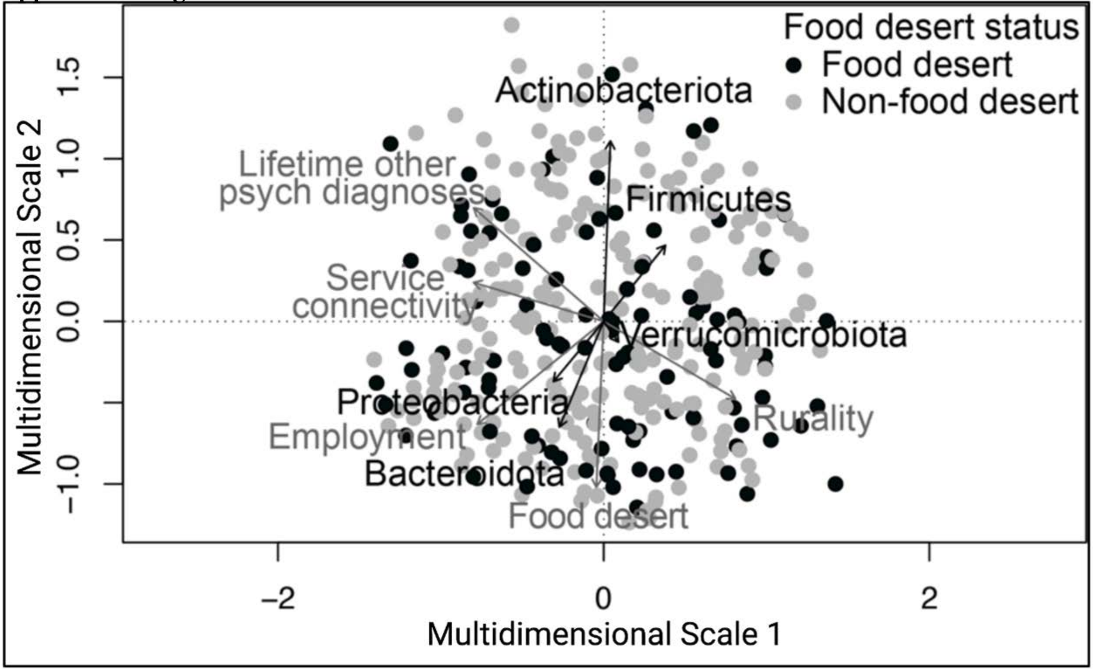

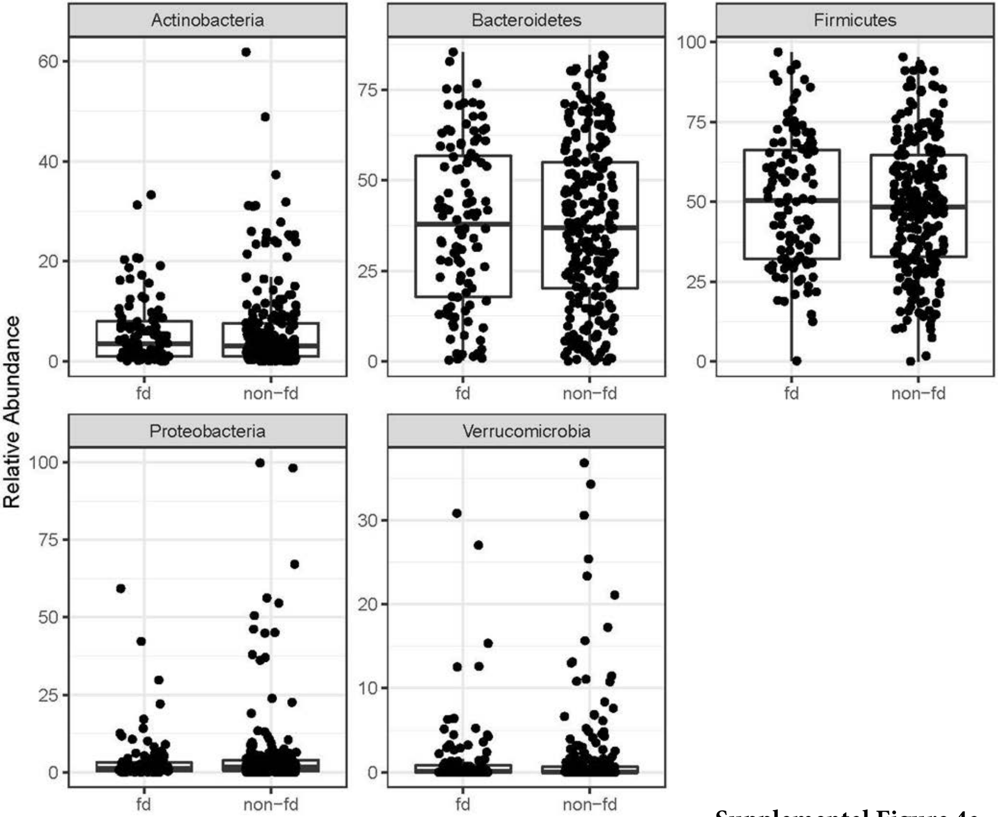

Supplemental Figure 4a

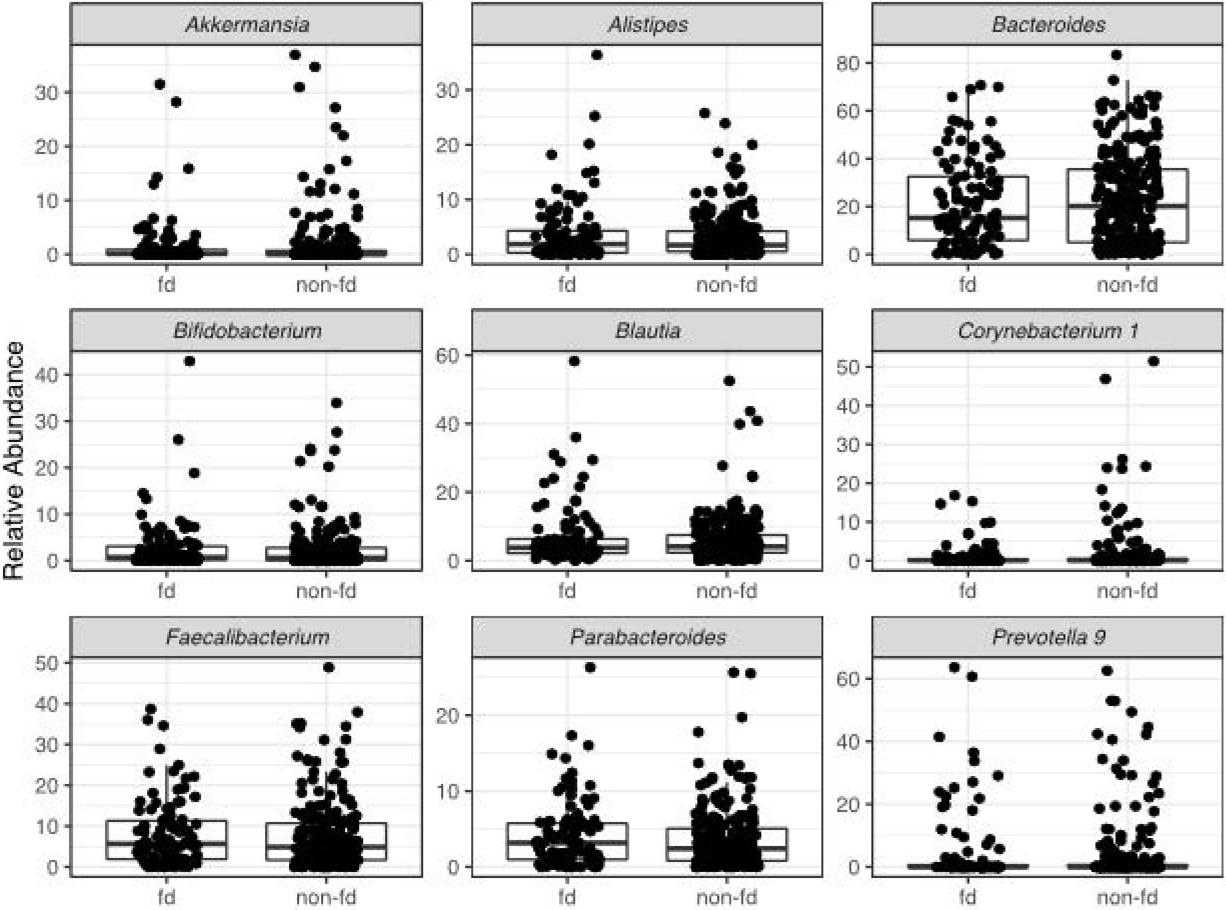

Supplemental Figure 4b
