# Supplementary material for: Food desert residence has limited impact on veteran fecal microbiome composition: a U.S. Veteran Microbiome Project study
Source: mSystems. 2023 Oct 24;8(6):e00717-23. doi: 10.1128/msystems.00717-23 (PMC10734509; doi:10.1128/msystems.00717-23)
Supplement: Supplemental Legends — Legends for supplemental figures. [file msystems.00717-23-s0002.docx]

**Supplemental Figure 1.** Alpha diversity of gut microbiome samples from participants in food desert and non-food desert groups, analyzed using (A) Observed Taxonomic Units (OTUS), (B) Shannon Diversity Index, and (C) Evenness.

**Supplemental Figure 2.** Principal Coordinate Analysis (PCoA) plot for (A) Weighted UniFrac and (B) UnWeighted UniFrac for food desert and non-food desert residents.

**Supplemental Figure 3.** Biplot of strongest correlates of the gut microbial community composition using unweighted UniFrac distance metric.

**Supplemental Figure 4a.** Relative abundances of the most prevalent phyla for food desert (fd) and non-food desert (non-fd) participants.

**Supplemental Figure 4b.** Relative abundances of the most prevalent genera for food desert (fd) and non-food desert (non-fd) participants.
